# Comparative real-world outcomes of tirzepatide vs semaglutide in patients with obesity and type2 diabetes: A retrospective propensity-matched cohort study

**DOI:** 10.1177/14791641261465360

**Published:** 2026-06-30

**Authors:** Abdul Qadeer, Marwah Bintay Khalid, Ridwan Syed, Samiksha Jain, Faiza Zakaria, Muhammad Huzaifa Ahmed Khan, Najam Gohar, Ashraf Shoukat, Ibrahim Mortada, Izhan Hamza, Afaq Motiwala, Muhammad Waheed Raja, Thomas Blackwell, Hani Jneid

**Affiliations:** 1 Department of Cardiovascular Medicine, University of Texas Medical Branch, Galveston, TX, USA; 2 Department of Medicine, 123683 Rawalpindi Medical University, Rawalpindi, Pakistan; 3 Department of Cardiovascular Medicine, The University of Texas Medical Branch, Galveston, TX, USA; 4 76093 Department of Medicine, Mobile Infirmary Medical Center, Mobile, AL, USA; 5 Department of Internal Medicine, Trinity Health Oakland/Wayne State University, Pontiac, Michigan, USA; 6 Department of Internal Medicine, Prime South GME Consortium Harlingen Medical Center, TX, USA; 7 Department of Internal Medicine, Ameer-ud-Din Medical College, Lahore, Pakistan; 8 619131 Department of Medicine, Nishtar Medical University, Multan, Pakistan; 9 Department of Internal Medicine, University of Texas Medical Branch, Galveston, TX, USA

**Keywords:** semaglutide, tirzepatide, obesity diabetes mellitus

## Abstract

**Background:**

With the increasing use of GLP-1 receptor agonists and dual GIP/GLP-1 agonists for managing obesity and type 2 diabetes, understanding their real-world effectiveness and safety is essential. This TriNetX analysis directly compares clinical outcomes among patients treated with tirzepatide vs semaglutide.

**Method:**

We utilized data from the TriNetX Research Network, identifying patients aged > 40 years or with obesity (“BMI ≥ 30 kg/m2”) and type 2 diabetes (HbA1c ≥ 6.5% or fasting glucose.≥ 125 mg/dL). Qualifying events were restricted to May 1, 2022, through November 21, 2024. We established two cohorts: one initiating tirzepatide and another semaglutide, ensuring each patient had at least three prescriptions and no prior exposure to the comparator drug or other GLP-1 receptor agonists. The index date was defined as the first co-occurrence of the obesity/diabetes criteria and the respective drug prescription. To ensure comparability, we performed 1:1 propensity matching, resulting in 47,804 patients in each cohort. Outcomes, including all-cause mortality, MACE, heart failure exacerbation, ischemic stroke/TIA, hospitalization/ED use, dementia, UTI, adverse Gastrointestinal (GI) effects, and changes in HbA1c, were assessed within a 1-year window after the index date.

**Results:**

Our matched cohort had a mean age of 75 years, with 45% male patients and 74% identified as white. Patients treated with tirzepatide experienced a significantly lower incidence of MACE at “(“3.7% vs 4.1% (RR 0.918, 95% CI 0.862-0.978). All-cause mortality was also lower with tirzepatide (0.2% vs. 0.4%; Risk Ratio 0.436, 95% CI 0.338-0.562). The tirzapetide group achieved better glycemic control with a lower mean HbA1c (6.565% vs. 6.848%; p < 0.001) during the follow-up. There was no significant difference in heart failure exacerbation or UTI incidence. GI side effects were slightly less frequent in the tirzepatide cohort (9.8% vs. 10.2%; Risk Ratio 0.959, 95% CI 0.924-0.997), while hospitalization or emergency visits were comparable between the two groups.

**Conclusion:**

In this propensity matched cohort of patients with obesity and type 2 diabetes, tirzepatide demonstrated improved cardiometabolic outcomes compared to semaglutide, including lower all-cause mortality and lower HbA1c. These findings support the cardiovascular safety and efficacy of tirzapetide and highlight the need for further studies to evaluate its effectiveness in broader clinical populations.

## 1. Introduction

Semaglutide, a long-acting GLP-1 receptor agonist, has been extensively studied in patients with T2DM and obesity. Large randomized controlled trials have shown that semaglutide produces significant reductions in HbA1c and substantial, sustained weight loss compared with placebo and other antidiabetic agents.^1^ In addition to its metabolic benefits, semaglutide has demonstrated cardiovascular risk reduction in high-risk patients, further strengthening its role in contemporary diabetes management.^2^

More recently, dual incretin receptor agonists have gained attention for their enhanced metabolic effects. Tirzepatide is a novel agent that simultaneously activates glucose-dependent insulinotropic polypeptide (GIP) and GLP-1 receptors, offering a complementary and potentially synergistic mechanism of action.^3^ By engaging both pathways, tirzepatide improves insulin sensitivity, enhances glycemic control, and exerts profound effects on appetite regulation and energy balance.

Clinical trials have demonstrated that tirzepatide leads to greater reductions in HbA1c and body weight compared with selective GLP-1 receptor agonists, including semaglutide, in patients with T2DM.^4^ These findings suggest that dual GIP/GLP-1 receptor agonism may provide superior metabolic outcomes, particularly in individuals with significant obesity or inadequate response to monotherapy GLP-1 receptor agonists.

Although GLP-1 receptor agonists such as semaglutide are well established and widely used, the long-term comparative effectiveness of dual-action agents like tirzepatide remain incompletely defined. Direct head-to-head comparisons are limited, and existing literature may not fully reflect the efficacy, tolerability, and clinical outcomes across diverse patient populations. Our study aims to systematically evaluate and compare the clinical outcomes for patients wit obesit an DM- 2 treated with GLP-1 receptor agonists versus dual GIP/GLP-1 receptor agonists.

## 2. Methods

### 2.1 Data source and study design

This study was designed as a retrospective, multicenter cohort analysis utilizing the TriNetX Research Network (TriNetX LLC, Cambridge, MA, USA). The analysis was performed on January 5, 2026. TriNetX is a global federated health research platform that provides access to de-identified electronic health records (EHRs) from participating academic medical centers, community hospitals, and specialty care organizations across the United States. The TriNetX Research Network used for this study included data from over 100 healthcare organizations. The database contains longitudinal clinical information, including demographics, diagnoses, procedures, medications, laboratory values, and healthcare utilization, harmonized using standardized clinical terminologies such as ICD-10-CM/PCS, CPT, RxNorm, and LOINC. The study period extended from May 1, 2022, through January 4, 2025, allowing for evaluation of contemporary prescribing patterns and clinical outcomes associated with glucagon-like peptide-1 (GLP-1) receptor agonists and dual GLP-1/glucose-dependent insulinotropic polypeptide (GIP) receptor agonists. During the study period, tirzepatide was approved for type 2 diabetes mellitus before being approved for chronic weight management. Therefore, medication exposure was defined based on prescription records rather than labeled regulatory indication. Because the TriNetX platform does not reliably capture prescribing indication, analyses reflect real-world use irrespective of formal regulatory indication. However, all included patients had documented obesity and type 2 diabetes mellitus before treatment initiation, ensuring clinical relevance to both metabolic and weight-related use. Because the TriNetX platform provides only de-identified data and is compliant with the Health Insurance Portability and Accountability Act (HIPAA) Privacy Rule (§164.514), this study was exempt from institutional review board approval. The study was conducted in accordance with the Strengthening the Reporting of Observational Studies in Epidemiology (STROBE) guidelines and adhered to TriNetX publication standards.

### 2.2 Population: Eligibility and exclusion criteria

**Figure 1.**
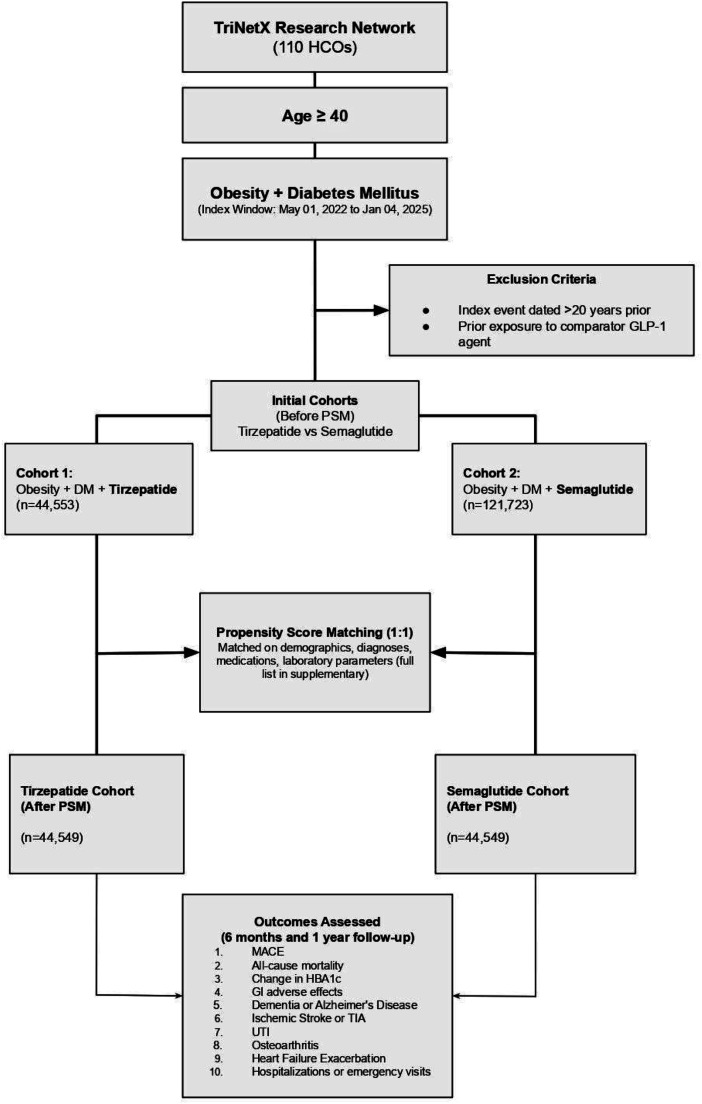
Flowchart of Patient Selection and Propensity Score Matched Cohorts of Tirzepatide vs Semaglutide.

The study population included adult patients aged ≥40 years with documented diagnoses of obesity and type 2 diabetes mellitus (T2DM). The age threshold of 40 years was selected to enrich the cohort for individuals at meaningful cardiometabolic risk and to improve event capture for cardiovascular and healthcare utilization outcomes during follow-up. This cutoff also reduces heterogeneity associated with early-onset obesity and diabetes phenotypes, which may differ in ≥30 kg/m^2^ or a recorded ICD-10-CM diagnosis of overweight or obesity (E66), reflecting clinician-documented weight status within the electronic health record. T2DM was identified using ICD-10-CM codes (E08–E13) and/or laboratory criteria, including hemoglobin A1c ≥6.5% or fasting plasma glucose ≥125 mg/dL. Eligible patients were those who initiated treatment with either tirzepatide or semaglutide between May 1, 2022, and January 4, 2025. To ensure true treatment exposure, inclusion required at least three recorded prescriptions of the index medication. The date of the first qualifying prescription, occurring on or after documentation of obesity and T2DM, was defined as the index date. Two exposure cohorts were constructed^1^: patients initiating tirzepatide and^2^ patients initiating semaglutide. Semaglutide exposure included all formulations recorded in the electronic health record. The TriNetX platform does not consistently distinguish between oral and subcutaneous formulations across all contributing healthcare organizations. Therefore, analyses reflect overall real-world semaglutide use irrespective of route of administration. However, subcutaneous formulations are expected to predominate given prescribing patterns during the study period. Patients were excluded if they had prior exposure to the comparator agent or other glucagon-like peptide-1 receptor agonists (including liraglutide, dulaglutide, or exenatide) before the index date. Additional exclusion criteria included incomplete demographics or follow-up data. The patient identification and cohort selection process is summarized in Figure 1. Outcomes were assessed at two prespecified follow-up intervals following the index date: 6 months (180 days) and 1 year (365 days). disease trajectory and treatment response. Obesity status was identified using either measured BMI.

### 2.3 Baseline characteristics and propensity score matching

To minimize confounding and approximate randomized treatment allocation, propensity score matching (PSM) was performed. Propensity scores were generated using logistic regression models incorporating a comprehensive set of baseline covariates, including demographics (age, sex, race, and ethnicity), comorbid conditions, prior medication use, and available baseline laboratory values. Matching was conducted in a 1:1 ratio using a nearest-neighbor approach with a caliper width of 0.1 standard deviations of the logit of the propensity score, without replacement. After matching, both cohorts consisted of 44,549 patients, ensuring equal sample sizes for comparative analyses. Baseline demographic, clinical, and laboratory characteristics before and after matching are presented in Table 1. Covariate balance between cohorts was evaluated using standardized mean differences (SMDs), with values <0.10 considered indicative of adequate balance. Visual inspection of propensity score density plots was used to confirm overlap and comparability between matched cohorts. Separate propensity-matched cohorts were constructed for the 6-month and 1-year analyses to ensure balance across distinct follow-up durations.

**Table 1.** Baseline characteristics of study subjects (before and after Propensity score matching).

​	Before matching			After matching		
	Tirzepatide group (n= 44,553)	Semaglutide group (n= 121,723)	Std diff	Tirzepatide group(n= 44,549)	Semaglutide group(n= 44,549)	Std diff
**Age at index**						
Mean±SD	57.8 +/- 10.4	59.4 +/- 10.7	0.152	57.8 +/- 10.4	57.9 +/- 10.6	0.004
**Gender, n (%)**						
Male	20,102 (45.1)	55,634 (45.7)	0.012	20,101 (45.1)	20,203 (45.4)	0.005
Female	24,441 (54.9)	66,062 (54.3)	0.012	24,438 (54.9)	24,338 (54.6)	0.005
**Race, n (%)**						
White	32,982 (74.0)	82,320 (67.6)	0.141	32,978 (74.0)	33,366 (74.9)	0.020
Black or African American	6,940 (15.6)	23,993 (19.7)	0.109	6,940 (15.6)	6,694 (15.0)	0.015
**Social economic status**						
Health hazards related to socioeconomic circumstances	1,735 (3.9)	5,798 (4.8)	0.043	1,735 (3.9)	1,698 (3.8)	0.004
**Comorbidities**						
Essential hypertension	32,758 (73.5)	96,190 (79.0)	0.130	32,757 (73.5)	32,878 (73.8)	0.006
Acute myocardial infarction	1,687 (3.8)	6,453 (5.3)	0.073	1,687 (3.8)	1,606 (3.6)	0.010
Cerebral infarction	1,495 (3.4)	5,190 (4.3)	0.047	1,495 (3.4)	1,444 (3.2)	0.006
Atrial fibrillation and flutter	3,358 (7.5)	10,740 (8.8)	0.047	3,358 (7.5)	3,265 (7.3)	0.008
Obstructive sleep apnea	14,916 (33.5)	40,093 (32.9)	0.011	14,915 (33.5)	14,857 (33.3)	0.003
Disorders of lipoprotein metabolism and other lipidemias	32,107 (72.1)	94,861 (77.9)	0.136	32,107 (72.1)	32,277 (72.5)	0.009
Type 2 diabetes mellitus	34,810 (78.1)	104,174 (85.6)	0.194	34,810 (78.1)	35,059 (78.7)	0.014
Heart failure	4,066 (9.1)	14,359 (11.8)	0.087	4,066 (9.1)	3,874 (8.7)	0.015
**Medication**						
Metoprolol	10,480 (23.5)	33,191 (27.3)	0.086	10,479 (23.5)	10,178 (22.8)	0.016
Spironolactone	3,023 (6.8)	9,556 (7.9)	0.041	3,023 (6.8)	2,886 (6.5)	0.012
Dapagliflozin	2,222 (5.0)	8,092 (6.6)	0.071	2,222 (5.0)	2,103 (4.7)	0.012
Metformin	23,779 (53.4)	77,575 (63.7)	0.211	23,779 (53.4)	24,001 (53.9)	0.010
INSULIN	13,261 (29.8)	45,012 (37.0)	0.153	13,261 (29.8)	12,926 (29.0)	0.017
**Laboratory,** Mean±SD						
BMI	38.3 +/- 7.1	37.5 +/- 6.8	0.115	38.3 +/- 7.1	37.9 +/- 6.9	0.055
LDL Cholesterol	95.8 +/-37.5	91.5 +/- 37.3	0.113	95.8 +/- 37.5	94.2 +/- 37.0	0.044
Hemoglobin A1c	7.2 +/- 1.7	7.3 +/- 1.7	0.101	7.2 +/- 1.7	7.2 +/- 1.7	0.044
Blood Pressure, Systolic	132.0 +/- 17.4	131.6 +/- 17.5	0.021	132.0 +/- 17.4	131.5 +/- 17.3	0.028
Blood Pressure, Diastolic	78.2 +/- 11.0	77.5 +/- 11.1	0.066	78.2 +/- 11.0	78.1 +/- 11.1	0.012

### 2.4 Study endpoints

Study outcomes were prespecified and categorized into metabolic, cardiovascular, renal, gastrointestinal, neurologic, musculoskeletal, infection-related, and healthcare utilization endpoints. The primary metabolic outcome was glycemic control, assessed using the most recent HbA1c within the follow-up. Secondary metabolic outcomes included weight-related measures, such as body mass index, where available. Cardiovascular outcomes included major adverse cardiovascular events (MACE), defined as a composite of acute myocardial infarction and ischemic stroke. Individual cardiovascular endpoints, including myocardial infarction, ischemic stroke or transient ischemic attack, heart failure exacerbation, and all-cause mortality, were also evaluated separately. Secondary outcomes included gastrointestinal adverse events and selected neurologic and musculoskeletal diagnoses, along with a prespecified falsification endpoint, as defined in Appendix C. Healthcare utilization outcomes included emergency department visits and hospitalizations. Dementia, osteoarthritis, and urinary tract infections were included as exploratory safety endpoints to assess broader health status and potential residual confounding, as no established mechanistic association with incretin-based therapies exists. Metabolic and laboratory outcomes were primarily emphasized at 6 months, while cardiovascular, safety, and healthcare utilization outcomes were evaluated at both 6 months and 1 year. Detailed outcome definitions are provided in Appendix C.

### 2.5 Statistical Analysis

Baseline characteristics were summarized using descriptive statistics and compared between cohorts before and after PSM. Continuous variables were reported as means with standard deviations, and categorical variables were summarized as counts and percentages. Clinical outcomes were evaluated using the TriNetX Compare Outcomes framework. Binary outcomes were analyzed using measures of association, including risk differences, risk ratios (RRs), and odds ratios (ORs), each reported with corresponding 95% confidence intervals (CIs). Time-to- event outcomes were assessed using Kaplan–Meier survival analyses with log-rank testing, and hazard ratios (HRs) with 95% CIs were estimated using Cox proportional hazards models. All analyses were performed within the TriNetX Analytics Platform using predefined index events and follow-up windows extending to 180 days for 6-month analyses and 365 days for 1-year analyses. Where applicable, supplementary figures were generated using R software (version 4.3.2). Statistical significance was defined as a two-sided p-value of less than 0.05.

## 3. Results

### 3.1 Study population

Our matched cohort had a mean age of 75 years, with 45% male patients and 74% identifying as white. After applying eligibility criteria and 1:1 propensity score matching, a total of 44,549 patients with obesity or diabetes mellitus were included in each respective cohort from the TriNetx US Collaborative Research Network: those treated with tirzepatide and those treated with semaglutide (Table 1). Qualifying clinical events occurred between May 1, 2022 and January 4, 2025 (Figure 1).

### 3.2 Population characteristics

Before propensity score matching, the Tirzepatide and Semglutide cohorts displayed several meaningful differences in various characteristics (Table 1). Patients in the Tirzepatide cohort were younger than those treated with Semaglutide (57.8 +/- 10.4 vs. 59.4 +/- 10.4, p<0.001) and consisted of a comparable sex distribution of females and males respectively. (55% vs. 54.3%, p<0.001). The white race was predominant in both cohorts (74% in Tirzepatide cohort vs. 67.6% in Semaglutide cohort, p<0.001). PSM effectively balanced these differences between the two cohorts.

### 3.3 Primary outcomes

The primary outcome was assessed at 6 months and 1 year (Tables 3 and Supplementary Table 1). The Tirzepatide cohort had a significantly lower risk of MACE at both the 6 month and 1 year mark, with greater absolute difference observed at 1 year Tirzepatide (3.4%) to Semaglutide (3.9%) (RR 0.87, 95% CI: 0.81-0.93, p<0.001) (Table 3; Supplementary Figure 2). All cause mortality was also significantly lower for the Tirzepatide cohort at both 6 months and 1 year follow ups. At 1 year, mortality occurred in.

76 patients treated with Tirzepatide and 181 patients treated with Semaglutide, corresponding to event rates of 0.1% vs. 0.2%; Risk Ratio 0.42, 95% CI: 0.32-0.55, p<0.001 (Supplementary Table 1 and Figure 2). Kaplan-Meier analysis demonstrated a consistent separation of event curves favoring tirzepatide for both MACE and all-cause mortality at the 1 year follow-up period (Supplementary Figure 2).

### 3.4 Secondary outcomes

Secondary outcomes were evaluated at 6 months and 1 year (Table 2 and Supplementary Table 1). At both time points, Tirzepatide was associated with significantly lower HbA1c levels when compared to Semaglutide. At 1 year being the largest difference (6.55% vs 6.82%, p<0.001) (Table 2).

**Table 2.** Change in HbA1c at 6 Months and 1 Year of follow-up after 1:1 propensity score matching between tirzepatide and semaglutide cohorts.

Outcome	Timepoint	Patients with outcomes		Mean	Standard deviation
		Tirzepatide (n = 44,549)	Semaglutide (n = 44,549)		
Change in Hba1c	6 months	24,083	24,885	6.71 vs 6.95	1.32 vs 1.41
​	1 Year	32,927	33,636	6.55 vs 6.82	1.29 vs 1.39

Moreover, at 1 year, UTIs were significantly less frequent in the tirzepatide cohort than the semaglutide cohort (RR 0.89, 95% CI 0.84-0.95, P<0.001). GI side effects were reported to be slightly less frequent in the Tirzepatide cohort at the 1 year mark (9.7% vs 10.0% RR 0.96, 95% CI: 0.93-1.00, p = 0.065) (Table 3). There was no significant difference in heart failure exacerbations, hospitalization, and emergency visits.

**Table 3.** Clinical outcomes at 1 Year of follow up after 1:1 propensity score matching.

Outcomes	Timepoint	Patients with outcomes		Risk ratio (95% CI)	p-value	Hazard ratio (95% CI)	p-value
		Tirzepatide (n = 44,549)	Semaglutide (n = 44,549)				
MACE	1 year	1,498	1,722	0.87 (0.81–0.93)	<0.001	0.87 (0.81–0.93)	<0.001
All-cause mortality	1 year	76	181	0.42 (0.32–0.55)	<0.001	0.48 (0.31–0.74)	0.001
GI adverse effects	1 year	4,302	4,466	0.96 (0.93–1.00)	0.065	0.985 (0.95-1.03)	0.477
Ischemic Stroke or TIA	1 year	998	1,074	0.929 (0.85-1.01)	0.091	0.947 (0.86-1.03)	0.211
UTI	1 year	1,856	2,084	0.891 (0.84-0.95)	<0.001	0.909 (0.85- 0.97)	0.003
Heart Failure Exacerbation	1 year	14,217	14,238	0.999 (0.98-1.02)	0.880	1.021 (0.99-1.05)	0.086
Hospitalizations or emergency visits	1 year	13,660	13,793	0.990 (0.97-1.01)	0.335	1.011 (0.99-1.04)	0.343

Matched cohorts: Tirzepatide (n=44,549) vs Semaglutide (n=44,549). Abbreviations: MACE, major adverse cardiovascular events; TIA, Transient Ischemic Attack; UTI, Urinary Tract Infection. Hazard ratios were derived from Cox proportional hazards models. Risk ratios represent cumulative incidence at the specified timepoint.

### 3.5 Cox proportional hazard model

Cox proportional model demonstrated a lower hazard for all cause mortality among patients treated with Tirzepatide compared to those treated with Semaglutide (1 year follow-up - HR 0.48, 95% CI 0.31-0.74; p = 0.001) (Tables 3 and Supplementary Table 1; Supplementary Figure 2). A statistical reduction in UTIs was observed in the tirzepatide cohort (HR: 0.91, 95% CI: 0.85-0.97). Similarly, patients treated with tirzapetide had a lower hazard for MACE compared to those treated with semaglutide, but this difference reached statistical significance only at 1 year follow up interval (HR 0.87, 95% 0.81-0.93, p<0.001) (Table 4). Gastrointestinal adverse events (HR 0.96, 95% CI 0.93-1.00; p =0.065), ischemic stroke/TIA (HR 0.93, 95% CI 0.65-1.01; p = 0.091), heart failure exacerbation(HR 1.00, 95% CI 0.98-1.02; p = 0.88), and hospitalizations/emergency (HR 0.99, 95% CI 0.97- 1.01; p = 0.0335) in between the groups were not significant. (Tables 3 and Supplementary table 1).

Kaplan-Meier survival curves were consistent with Cox proportional hazard modeling exhibiting lower cumulative events for MACE and all-cause mortality among patients treated with tirzepatide than compared with those who received semaglutide across the 1-year follow-up (Supplementary Figure 2).

### 3.6 Sensitivity analysis

Sensitive analyses were conducted with predetermined falsification endpoints to check for potential confounding. Osteoarthritis did not differ between groups at 6 months (RR 0.96, p=0.150) or at 1 year (RR 0.96, p=0.065. However, UTIs demonstrated lower frequency in the tirzepatide cohorts at both 6 months (RR=0.88, p=0.002) and 1 year (RR 0.89, p <0.001).

## 4. Discussion

In our real world propensity matched cohort of patients with obesity and DM-2 (Figure 1), treatment with tirzapetide was associated with lower observed all cause mortality and lower MACE, when compared to patients treated with semaglutide. Patients treated with tirzapetide also demonstrated improved glycemic control, as reflected by lower mean A1c as well as lower GI adverse effects. However there was no difference in heart failure exacerbations or urinary tract infection (UTI) incidence between the two groups.

Multiple trials have established the efficacy of both tirzepatide (a dual incretin agonist of the glucagon-like peptide-1 (GLP-1) and glucose-dependent insulinotropic poplypeptide (GIP) receptors) and selective GLP-1 receptor agonists, such as semaglutide, in reducing major adverse cardiovascular events.^5^ Previously, tirzepatide was observed to be equally effective to dulaglutide in respect to a composite of death of cardiovascular causes, myocardial infarction (MI) or stroke.^6^ Similarly, comparable cardiovascular benefit has been noted with tirzepatide and semaglutide.^7^ The discrepancy observed in their results compared to our findings could be attributed to variability in demographics, comorbidities, and cardiovascular risk factors of the patient cohorts. Comparable to our results, lower incidence of MACEs have been noted in patients with T2DM and obstructive sleep apnea receiving tirzepatide, in comparison to patients receiving semaglutide and liraglutide.^8^ Lower outcomes of acute MI, ischemic stroke or all-cause mortality has also been identified in patients receiving tirzepatide in comparison to GLP-1 RAs.^9^

Recent meta-analyses have reported significant reduction in MACE, and all-cause mortality with GLP-1 RA or GIP/GLP-1 RA. In addition, significant reduction of MI and non fatal MI events compared to placebo has been observed with these agents.^5^ Previously, reduced risk of MACE with liraglutide use has been established in high risk patients with T2DM, in addition to significant reduction in death from cardiovascular causes, or from any cause.^10^ Similarly, semaglutide, and dulaglutide have shown benefit in significantly reducing the risk of MACEs.^2,11^ When compared to placebo or insulin regimens, tirzepatide has demonstrated similar efficacy to insulin glargine,^12^ with the highest dose of tirzepatide associated with fewer total MACE events than insulin.^13^ Tirzepatide has been beneficial in contributing to fewer heart failure hospitalizations and urgent visits in patients with heart failure with preserved ejection fraction (HFpEF) and obesity, thereby reducing the endpoint of cardiovascular death or worsening heart failure on tirzepatide in comparison to placebo.^14^

Apart from glycemic control and weight loss properties, the mechanism of GLP-1/GIP RAs that protects from cardiovascular events is multifaceted. GLP1R is present within the atrial and ventricular cardiomyocytes, with highest GLP1R expression in the sinoatrial (SA) node. In comparison, GIPR is expressed in the ventricular myocardium. Enhanced activity of these receptors therefore influences the myocardium and subsequently the cardiovascular system.^15^ Tirzepatide has been hypothesized to express greater for GIP than GLP-1 receptors. Therefore, its enhanced cardiac activity would likely be attributed to its GIP component.^16^ Previous studies have also emphasized the impact of tirzepatide on inhibition of TLR-4/NF-KB/NLRP3, and its consequent impact on left ventricular remodeling.^17^ Studies have noted human cardiac cells express a higher level of GIP receptors compared to GLP-1 receptors, which would support the role of tirzepatide in protecting the cardiac cells against cell death, fibrosis and hypertrophy.^15^

Importantly, GLP-1 RAs such as semaglutide have demonstrated efficacy in patients who had a BMI of ≥27 with preexisting cardiovascular disease, but no history of diabetes. A significant reduction in primary cardiovascular endpoint was observed. Therefore, GLP-1 RAs have been shown to extend cardiovascular benefit in patients with and without T2DM. In addition, REWIND trial also establishes the benefit of GLP-1 RAs in the primary prevention of cardiovascular disease.^18^ Further studies are warranted to evaluate similar efficacy of GLP-1/GIP RAs in patients without preexisting diabetes or cardiac disease.

In light of our findings, tirzepatide is an effective agent in patients with obesity and T2DM in contributing to lower incidence of cardiac events, and mortality in this population. These findings are important in directing the use of GLP-1/GIP RAs in patient populations with established cardiovascular risk factors. Further studies are warranted to evaluate whether tirzepatide would be of similar benefit in patients without T2DM. In addition, the existence of prior cardiovascular events in the cohort should be evaluated, to determine the efficacy of tirzepatide in primary prevention of cardiovascular disease.

## 5. Limitations

There are several limitations of this study. Due to the retrospective, observational nature of this study.Causal relationships between treatment exposure and outcomes cannot be established despite rigorous propensity-score matching.

Medication dosing information was not available in sufficient detail to account for differences in dose escalation, duration at maintenance dose, treatment adherence, temporary discontinuation, or incomplete titration. Both tirzepatide and semaglutide require gradual dose escalation, and individual variation in achieved dose intensity may have influenced clinical outcomes, glycemic control, and adverse event rates.

Although propensity matching balanced numerous baseline characteristics, the database could not fully capture factors that may affect cardiovascular and metabolic outcomes, including dietary habits, physical activity, weight-management interventions, smoking status, socioeconomic factors, healthcare access, and patient adherence to prescribed therapies.

The presence of prior and concurrent treatments are not accounted for. There may have been exposure to glucose-lowering agents, cardioprotective medications, lipid-lowering therapies, antihypertensive agents, and weight-loss interventions before or during follow-up, that may have contributed to the observed differences in outcomes.

Finally, patients prescribed tirzepatide may differ from those prescribed semaglutide in ways not fully captured by available clinical variables, including physician prescribing preferences, treatment selection biases, and temporal differences in drug therapy. Prospective randomized studies with detailed assessment of medication dosing, treatment adherence, and lifestyle factors are needed to confirm the associations observed in this analysis.

## Supplemental material

Supplemental material - Comparative real-world outcomes of tirzepatide vs semaglutide in patients with obesity and type2 diabetes: A retrospective propensity-matched cohort study Supplemental material for Comparative real-world outcomes of tirzepatide vs semaglutide in patients with obesity and type2 diabetes: A retrospective propensity-matched cohort study by Abdul Qadeer, Marwah Bintay Khalid, Ridwan Syed, Samiksha Jain, Faiza Zakaria, Muhammad Huzaifa Ahmed Khan, Najam Gohar, Ashraf Shoukat, Ibrahim Mortada, Izhan Hamza, Afaq Motiwala, Muhammad Waheed Raja, Thomas Blackwell and Hani Jneid in Diabetes & Vascular Disease Research.

## Data Availability

This is deidentified data available through trinetx.*
