# Supplementary material for: Comparative real-world outcomes of tirzepatide vs semaglutide in patients with obesity and type2 diabetes: A retrospective propensity-matched cohort study
Source: Diab Vasc Dis Res. 2026 Jun 30;23(3):14791641261465360. doi: 10.1177/14791641261465360 (PMC13319829; doi:10.1177/14791641261465360)
Supplement: Supplemental material - Comparative real-world outcomes of tirzepatide vs semaglutide in patients with obesity and type2 diabetes: A retrospective propensity-matched cohort study [file sj-pdf-1-dvr-10.1177_14791641261465360.pdf]

Comparative Real-world Outcomes of Tirzepatide Vs Semaglutide in Patients with Obesity and Type2 Diabetes: A Retrospective Propensity-matched Cohort Study

Corresponding author: Abdul Qadeer

Department of Cardiovascular Medicine, University of Texas Medical Branch, Galveston, TX,USA. Email: abqadeer@utmb.edu

Supplementary File

Supplementary Figure 1. Propensity Score Density Function - Before and After Matching

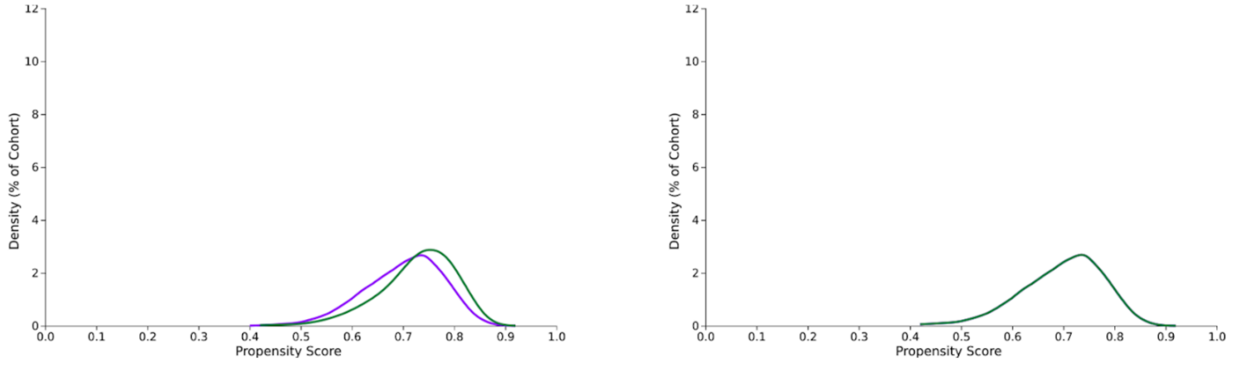

Supplementary Figure 2. Kaplan Meier Survival Analysis Plots for 1-Year Outcomes (Purple = Tirzepatide, Green = Semaglutide)

Hospitalizations or emergency visits

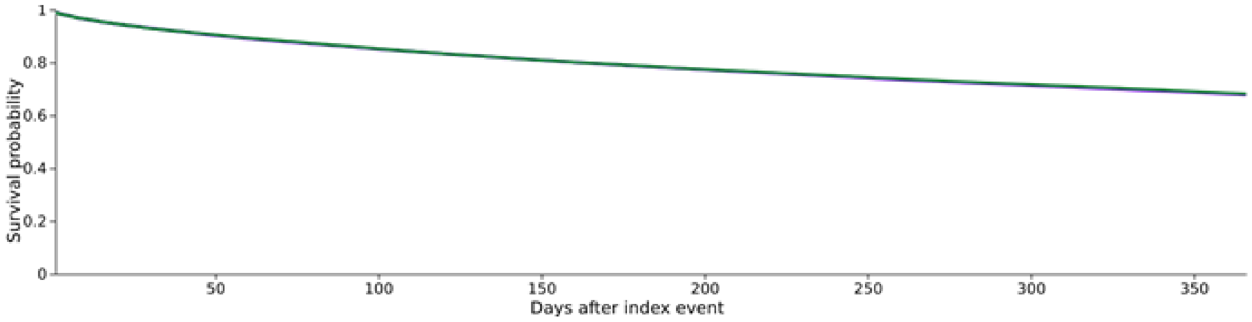

MACE

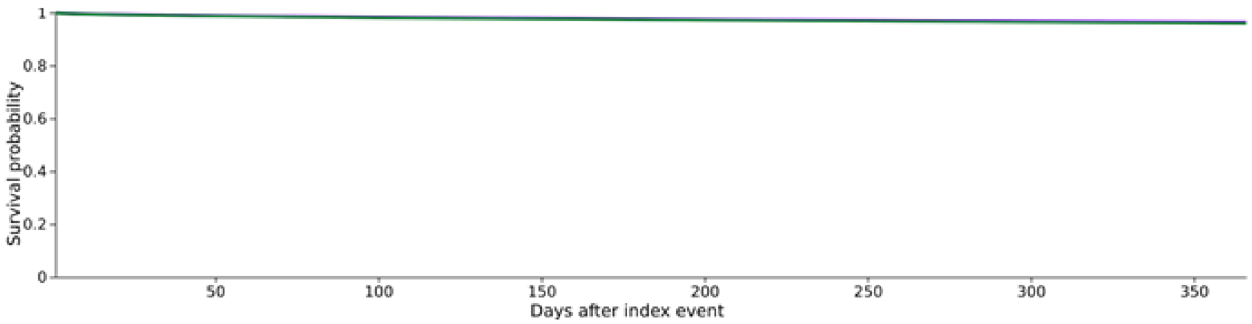

HF Exacerbation

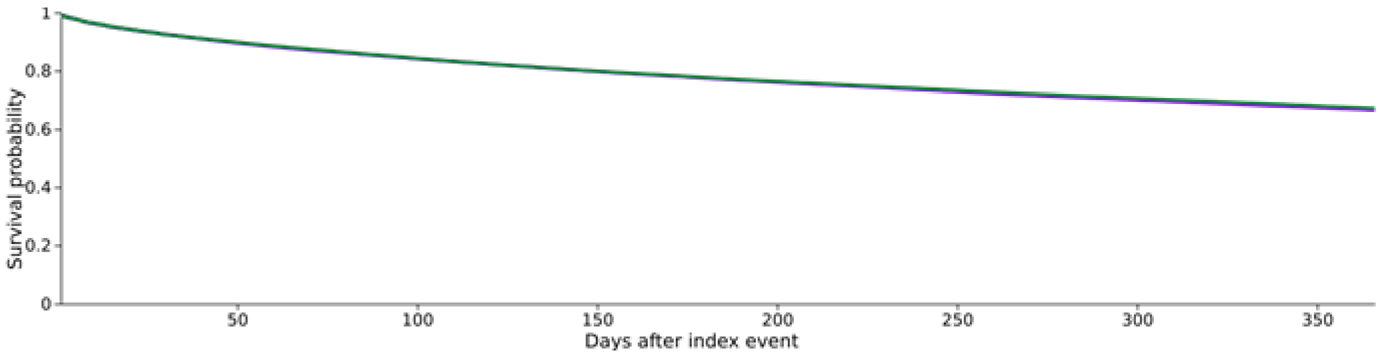

Dementia or Alzheimer's Disease

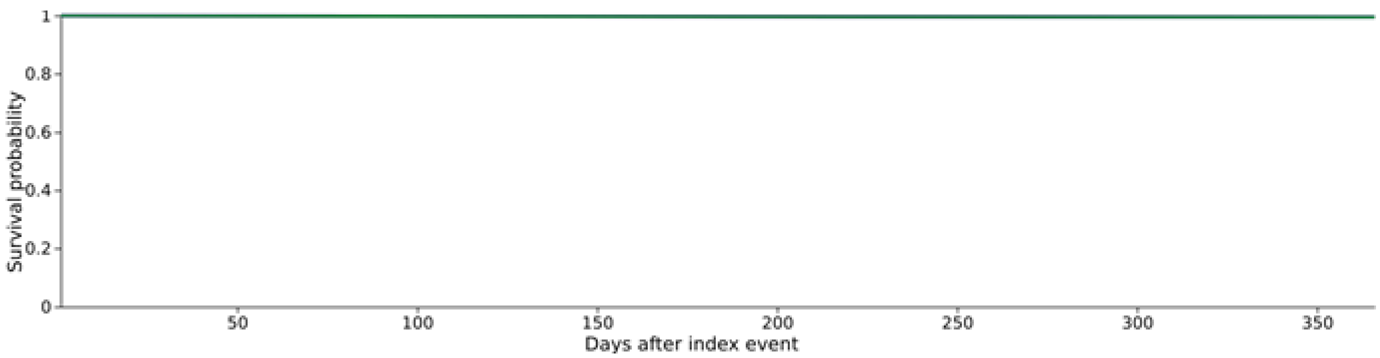

UTI

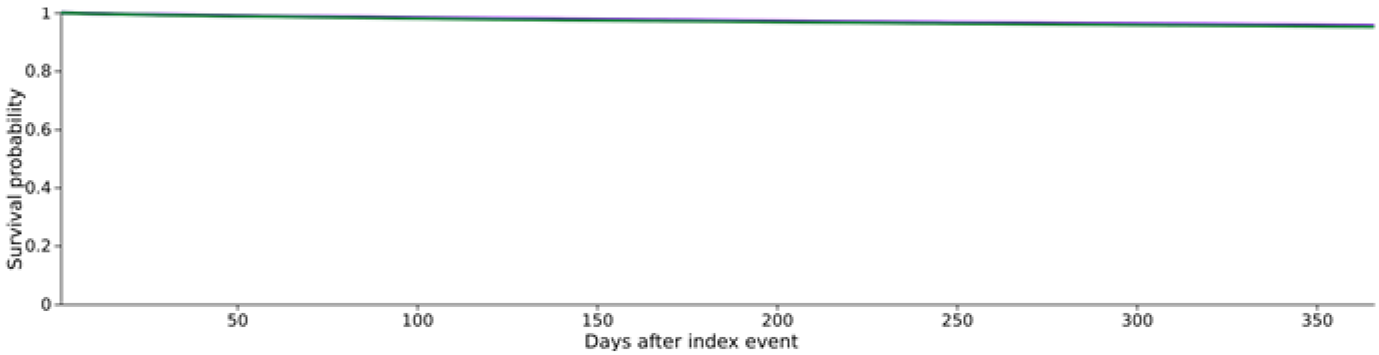

All-Cause Mortality

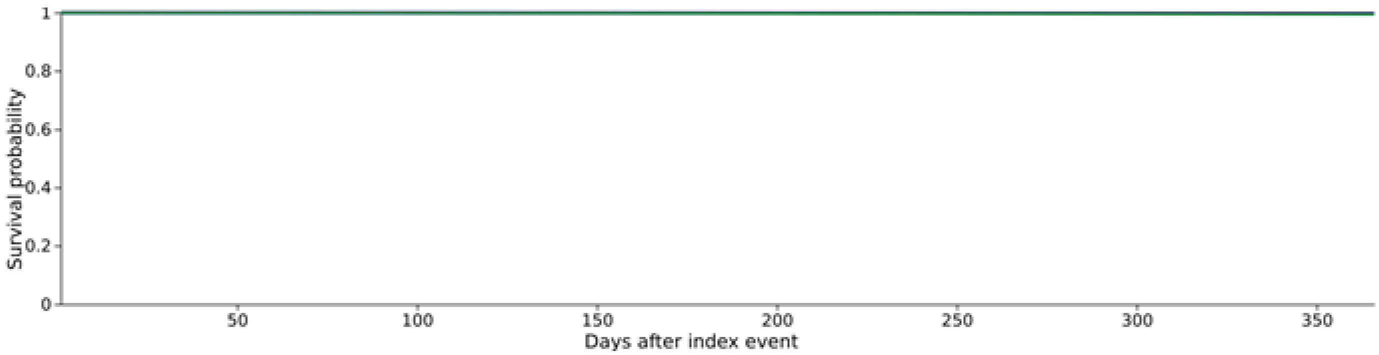

GI Side effects

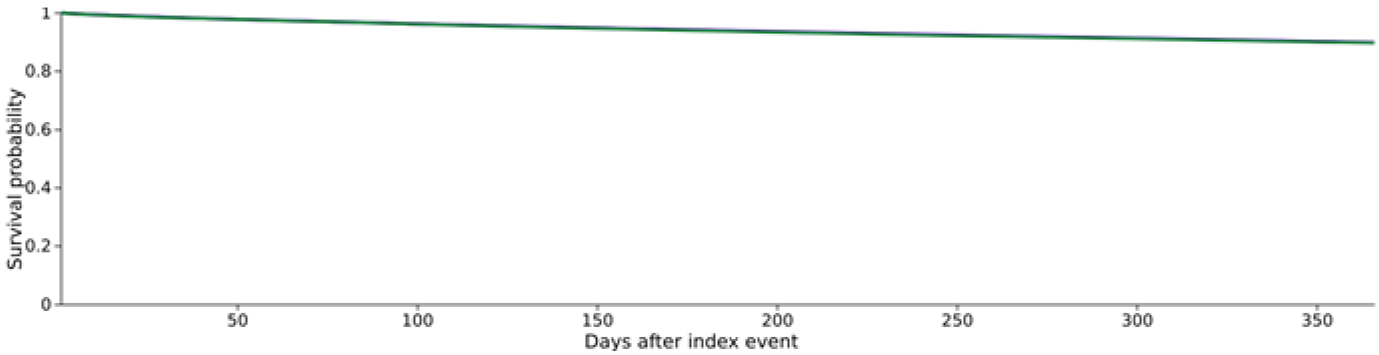

Ischemic Stroke/TIA

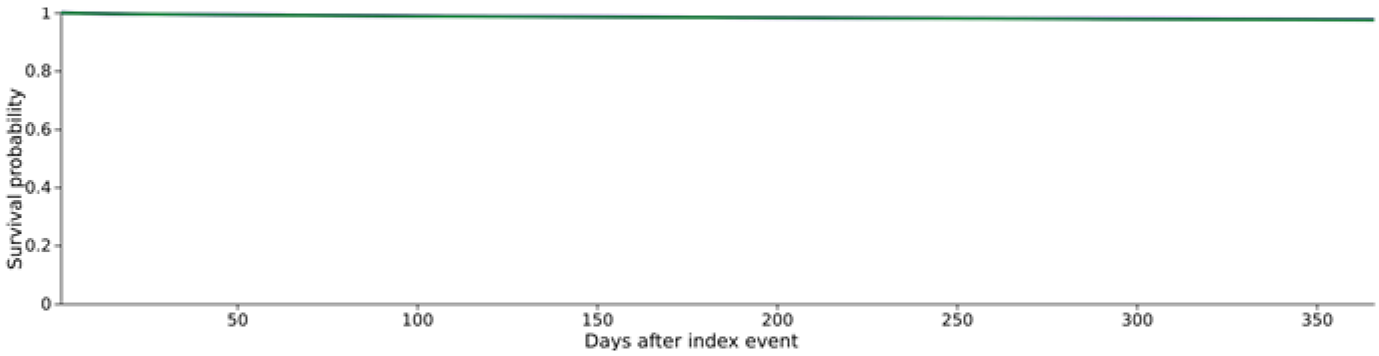

Osteoarthritis

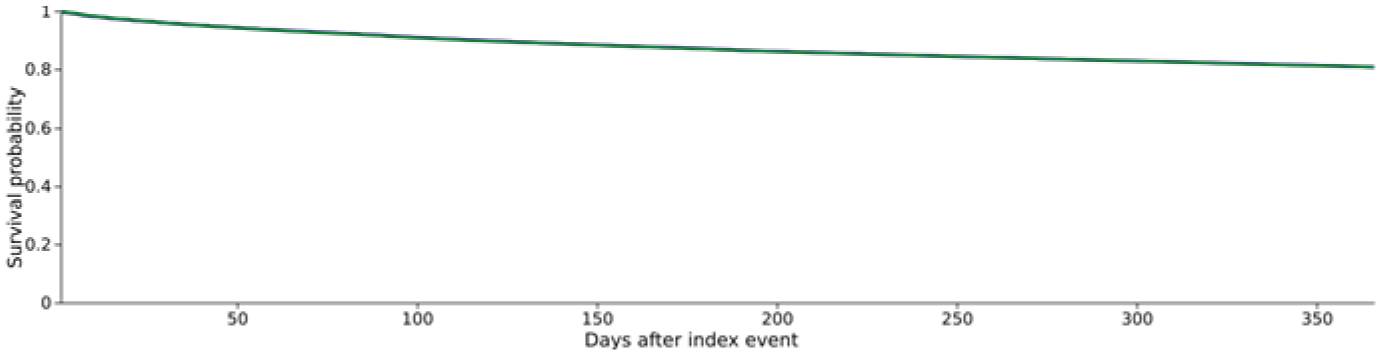

Supplementary Table 1 : Clinical Outcomes at 6 months of Follow up After 1:1 Propensity Score Matching.

Outcomes	Timepoint	Patients with Outcomes	Risk Ratio (95% CI)	p-value	Hazard Ratio (95% CI)	p-value

		Tirzepatide (n = 44,549)	Semaglutide (n = 44,549)				
MACE	6 months	1,029	1,127	0.913 (0.84-0.99)	0.033	0.917 (0.84-0.99)	0.046
All-cause mortality	6 months	30	63	0.476 (0.31-0.74)	0.001	0.482 (0.31-0.74)	0.001
GI adverse effects	6 months	2,615	2,717	0.962 (0.91-1.01)	0.150	0.969 (0.92-1.02)	0.250
Ischemic Stroke or TIA	6 months	700	718	0.975 (0.88-1.08)	0.630	0.981 (0.88-1.09)	0.711
UTI	6 months	1,141	1,292	0.883 (0.82-0.96)	0.002	0.888 (0.82-0.96)	0.003
Heart Failure Exacerbation	6 months	9,903	9,879	1.002 (0.98-1.03)	0.847	1.010 (0.98-1.04)	0.475
Hospitalizations or emergency visits	6 months	9,382	9,397	0.998 (0.97-1.02)	0.902	1.006 (0.98-1.04)	0.698

Matched cohorts: Tirzepatide (n=44,549) vs Semaglutide (n=44,549). Abbreviations: MACE, major adverse cardiovascular events; TIA, Transient Ischemic Attack; UTI, Urinary Tract Infection. Hazard ratios were derived from Cox proportional hazards models. Risk ratios represent cumulative incidence at the specified timepoint.
